# Sugammadex is associated with shorter hospital length of stay after open lobectomy for lung cancer: a retrospective observational study

**DOI:** 10.1186/s13019-021-01427-9

**Published:** 2021-03-23

**Authors:** Seung Won Song, Kyung Yeon Yoo, Yong Sung Ro, Taehee Pyeon, Hong-Beom Bae, Joungmin Kim

**Affiliations:** Department of Anesthesiology and Pain Medicine, Chonnam National University Medical School, Chonnam National University Hospital, 160, Baekseo-ro, Dong-gu, Gwangju, 501-746 South Korea

**Keywords:** Sugamadex, Pyridostigmine, Length of stay, Lobectomy

## Abstract

**Background:**

Sugammadex is associated with few postoperative complications. Postoperative pulmonary complications (PPC) are related to prolonged hospitalizations. Present study explored whether the use of sugammadex could reduce PPCs and thereby reduce hospital length of stay (LOS) after lung surgery.

**Methods:**

We reviewed the medical records of patients who underwent elective open lobectomy for lung cancer from January 2010 to December 2015. Patients were divided into the sugammadex group and pyridostigmine group.

The primary outcome was hospital LOS and secondary outcomes were postoperative complications and overall survival at 1 year. The cohort was subdivided into patients with and without prolonged LOS to explore the effects of sugammadex on outcomes in each group. Risk factors for LOS were determined via multivariate analyses. After propensity score matching, 127 patients were assigned to each group.

**Results:**

Median hospital LOS was shorter (10.0 vs. 12.0 days) and the incidence of postoperative atelectasis was lower (18.1 vs. 29.9%) in the sugammadex group. However, no significant difference in overall survival between the groups was seen over 1 year (hazard ratio, 0.967; 95% confidence interval, 0.363 to 2.577). Sugammadex was a predictor related to LOS (exponential coefficient 0.88; 95% CI 0.82–0.95).

**Conclusions:**

Our data suggest that sugammadex is a preferable agent for neuromuscular blockade (NMB) reversal than cholinesterase inhibitors in this patient population.

**Trial registration:**

This study registered in the Clinical Research Information Service of the Korea National Institute of Health (approval number: KCT0004735, Date of registration: 21 January 2020, Retrospectively registered).

## Background

Lung resection surgery is associated with a high incidence of postoperative pulmonary complications (PPCs), including prolonged air leak, atelectasis, pneumonia, empyema, and acute respiratory distress syndrome [1]. PPCs are associated with prolonged hospitalizations, admissions to the intensive care unit (ICU), and hospital re-admissions, as well as increased morbidity, mortality, and healthcare expenditure [2–6]. Residual neuromuscular blockade (NMB) after emergence from anesthesia can lead to pulmonary complications including hypoxia, pulmonary collapse, and acute respiratory failure [7, 8]. In addition, complete pharmacological reversal improves pulmonary outcomes by reducing the likelihood of residual neuromuscular blockade after anesthesia [9].

Anticholinesterases such as neostigmine or pyridostigmine are commonly used to reverse NMB during general anesthesia. Despite the use of relatively short-acting agents (e.g., rocuronium), which decrease the incidence of residual NMB, the incidence of residual NMB is reported to 82% at 6 min post administration of neostigmine [10]. And it has various cholinergic side effects including bradycardia, hypotension, bronchoconstriction, and airway secretion [11, 12]. Sugammadex is modified gamma-cyclodextrin compound that selectively binds to steroidal non-depolarizing neuromuscular blocker by forming a 1:1 complex. In contrast to anticholinesterase, it shows rapid and reliable neuromuscular block reversal [13]. It reduces postoperative complications and improves patient outcomes [14].

Hospital length of stay (LOS) is considered an important quality metric for recovery from surgery and anesthesia [15]. The length of time patients spend in hospital is a good representation of the amount of hospital resources utilized, such as bed utilization, staffing, and equipment [16]. To date, few studies have evaluated the relationship between neuromuscular block reversal with sugammadex and patient outcomes including LOS with controversial results in different surgeries [12, 17–19].

We hypothesized that there would be a difference in the LOS due to PPC when the NMB reversed with sugammadex compared pyridostigmine after open lobectomy for lung cancer. We investigated the relationship between reversal agent and LOS/postoperative complication rate/overall survival, and identified risk factors associated with prolonged LOS in these patients.

## Methods

### Study population and data collection

This study was approved by the institutional review board of Chonnam National University Hwasun Hospital (approval number: CNUHH-2019-207, 19 Dec. 2019) and registered in the Clinical Research Information Service of the Korea National Institute of Health (approval number: KCT0004735). Data was collected retrospectively by Seung Won Song from electronic patient medical records at Chonnam National University Hwasun Hospital. Patients 19 years of age or older and of American Society of Anesthesiologists (ASA) physical status I to III who underwent elective open lobectomy for lung cancer from January 2010 to December 2015 were included. Sugamadex was introduced in our hospital in 2013, and has been used for thoracic surgery since 2014. The information collection period was set to make the frequency of use of the two drugs similar. There are various surgical procedures for lung cancer, but only open lobectomy was included in the study in order to reduce the variance between the types of surgery. Excluded from analyses were patients who were paralyzed with neuromuscular blocker other than rocuronium (e.g., cisatracurium), reversed with an agent other than pyridostigmine or sugammadex (e.g., neostigmine due to its different pharmacokinetic features such as onset, duration, potency), transferred to ICU for recovery from anesthesia, or missing any medical records.

Patients were divided into sugammadex and pyridostigmine groups according to the type of reversal agent used during surgery. The following data were collected for the study. Preoperative data: age, sex, height, weight, body mass index (BMI), forced expiratory volume in 1 s (FEV1) / forced vital capacity (FVC), and preoperative comorbidities (including ASA physical status, diabetes mellitus [DM], hypertension [HTN], chronic kidney disease [CKD], heart failure [HF], coronary arterial disease [CAD], chronic obstructive pulmonary disease [COPD], and asthma). Intra-operative data: location of surgery (right or left), surgery time, agent used for neuromuscular block reversal. Postoperative data: LOS after surgery, duration of stay in postanesthesia care unit (PACU), postoperative complications (pyrexia, dyspnea, air leak > 5 days, atelectasis, pneumonia, mechanical ventilator use, hemodynamic instability, and ICU admission), and type of patient-controlled analgesia (epidural or intravenous). In-hospital standard medication regimen was used for pain control. In case of epidural catheter placement, fentanyl with chirocaine patient controlled analgesia (PCA) was used. Patients without epidural catheter were received intravenous fentanyl PCA. No additional block was performed.

Postoperative complications were examined by reviewing medical records. Pyrexia was defined as having a tympanic membrane temperature greater than 38.0 °C. Dyspnea was defined as presenting with complaints of feeling short of breath or showing blue-tinged fingers or lips and/or use of accessory muscles or chest muscles to breathe. Hemodynamic instability was defined as a fall in the systolic blood pressure below 90 mmHg with symptoms related to hypotension, including chest discomfort and altered consciousness level, requiring immediate pharmacological rescue (e.g., vasopressor or inotrope). Atelectasis and pneumonia were diagnosed based on a serial postoperative plain chest radiograph routinely checked until discharge.

The primary endpoint was the difference in LOS after open lung lobectomy between reversal with sugammadex and pyridostigmine. The secondary endpoints were differences in postoperative complications and overall survival between the two reversal agents. We performed additional analyses after dividing the cohorts into two subgroups (patients with prolonged LOS (LOS > 14 days) vs. those without (LOS ≤ 14 days)). Prolonged LOS was defined as hospitalization beyond 14 days, as suggested in a previous report [20]. Intra- and postoperative outcomes were compared between the two groups in each subgroup. Multivariate poisson and logistic regression using stepwise variable selection was used to identify perioperative risk factors associated with LOS.

### Statistical analysis

Continuous variables are presented as the mean ± standard deviation (SD) for normally distributed data or median (interquartile range, IQR) for non-normally distributed data and were compared using an unpaired Student *t*-test or Wilcoxon rank-sum test, as appropriate. Normality was verified based on the Shapiro-Wilk test or by inspecting histograms or Q-Q plots. Categorical variables are presented as numbers (percentage, %) and were compared using Pearson’s χ^2^ test or Fisher’s exact test. The Kaplan-Meier method was applied for analyses of discharge rate and survival rate [21, 22]. We investigated the outpatient visit to investigate the patient’s one-year survival. If there were any hospital visit history after 1 year from surgery, patient was assumed to survive after 1 year. But if there were any record declaring death of patient, we concluded that the patient was dead. The variables were compared using the log-rank test and the Cox proportional hazard ratio was estimated for survival analyses. *P* value < 0.05 was considered statistically significant; all tests were two-sided.

LOS is a naturally skewed distribution in most cohorts [16], so we constructed a multivariate Poisson regression model for LOS as a response variable to identify risk factors that increase the LOS. A multivariate logistic regression model was also constructed to identify risk factors associated with prolonged LOS. Covariates were classified into demographic predictors and intra- or postoperative predictors to construct two different models. Initially, a univariate regression was performed to screen covariates associated with the response variable. Covariates with a *p* value < 0.2 in univariate regression were included in the multivariate regression model. Final covariates were selected using the forward and backward stepwise elimination method based on Akaike Information Criterion. Exponential coefficients and their 95% confidence interval (CI) for the Poisson model and odds ratio (OR) and their 95% CI for the logistic model was estimated for each covariate in the final model.

Propensity score matching was performed to reduce potential selection bias. Covariates used in propensity score matching were as follows. Age, sex, ASA physical status, DM, HTN, CKD, HF, CAD, COPD, asthma, operation site, FEV1/FVC, operation time, BMI. Nearest neighbor method was used and ratio was 1:1. R code used in analysis was as follows. This approach estimated the probability of individuals receiving sugammadex as an agent for neuromuscular block reversal and allows for comparison with pyridostigmine-receiving patients with similar demographic and clinical characteristics. The score of each patient was calculated by estimating the probability to be assigned to each neuromuscular block reversal agent using multivariate logistic regression. The balance of the two groups was assessed based on standardized differences. All statistical analyses and tests were performed using R, a software environment for statistical computing (R version 3.6.0; The R Foundation for Statistical Computing, Vienna, Austria). Propensity score matching was performed using package MatchIt in R program (version 3.0.2).

## Results

**Fig. 1:**
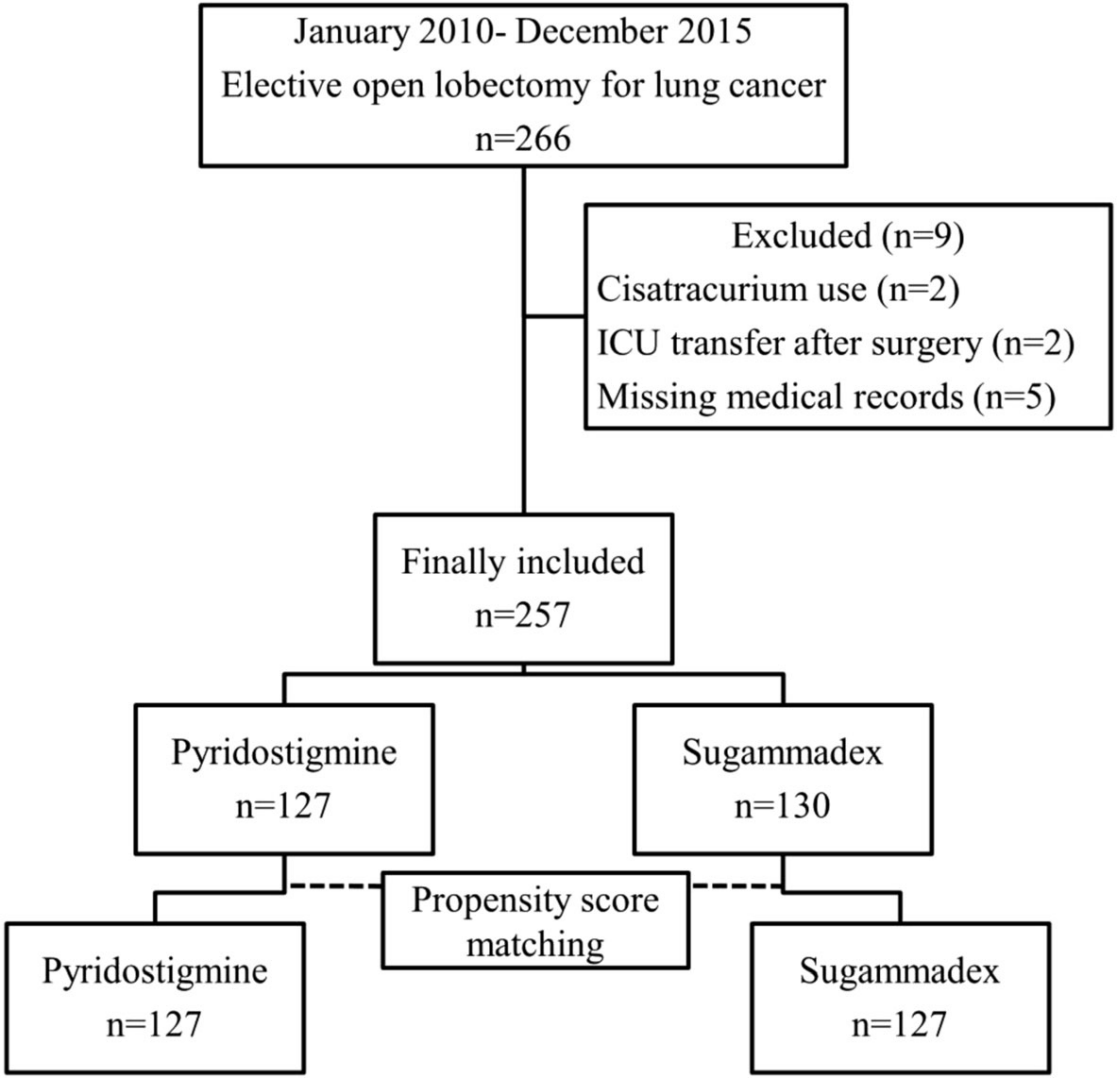
Flow chart of patient selection

During the study period, 266 patients underwent open lobectomy for lung cancer at our institution. Of those patients, 9 were excluded due to cisatracurium use, ICU transfer after surgery, or missing medical records. A total of 257 patients were enrolled; 127 patients received pyridostigmine (pyridostigmine group) and 130 patients received sugammadex (sugammadex group) for NMB reversal. After propensity score matching, 127 patients in each group were included in the final analyses (Fig. 1). Demographic and clinical characteristics at baseline are summarized in Table 1 and were comparable between the two groups.

**Table 1:** Demographic and clinical characteristics at baseline of matched cohort

Variable	Pyridostigmine (*n* = 127)	Sugammadex (*n* = 127)	*p* value
Age (year)	66.0 (59.5–71.0)	67.0 (60.0–72.0)	0.358
Male sex	92 (72.4)	96 (75.6)	0.668
Height (cm)	163.0 (159.0–168.5)	163.0 (158.0–168.0)	0.640
Weight (kg)	62.0 (56.0–67.0)	61.0 (56.0–69.5)	0.817
BMI (kg m^−2^)	23.1 (21.5–25.5)	23.5 (21.3–25.7)	0.445
ASA PS			0.238
I	14 (11.0)	19 (15.0)	
II	104 (81.9)	93 (73.2)	
III	9 (7.1)	15 (11.8)	
Diabetes mellitus	22 (17.3)	27 (21.3)	0.525
Hypertension	55 (43.3)	55 (43.3)	1.000
CKD	5 (3.9)	2 (1.6)	0.443
Heart failure	3 (2.4)	0 (0.0)	0.245
CAD	14 (11.0)	12 (9.4)	0.836
COPD	28 (22.0)	31 (24.4)	0.766
Asthma	3 (2.4)	0 (0.0)	0.245
Operation site (right)	85 (66.9)	76 (59.8)	0.297

Data are presented as n (%), median (interquartile range)

*BMI* Body mass index, *ASA PS* American Society of Anesthesiologists physical status, *CKD* Chronic kidney disease, *CAD* Coronary arterial disease, *COPD* Chronic obstructive pulmonary disease

The collected data included 4 thoracic surgeons and 5 anesthesiologists (excluding residents). The initial dose of rocuronium was 0.8–1.0 mg/kg. Pyridostigmine was 0.1–0.2 mg/kg and sugammadex was 1.5–2 mg/kg for reversal of NMB. The use of sugammadex continued after it was decided at the anesthesiology faculty meeting, which led to more intensive NMB during surgery. The degree of NMB during anesthesia was monitored using the MechanoSensor™ DatexOhmeda GE Healthcare NMT-EMG (Helsinki, Finland). However, it was excluded from the analysis due to inconsistent medical records. A double lumen endotracheal tube was used for one-lung ventilation during surgery, and the surgical approach for the affected area was a conventional posterior lateral thoracotomy in the lateral position. In the following cases, an anesthesiologist discussed with a thoracic surgeon to determine whether to extubate: Difficulty breathing before surgery, hypoxemia (blood oxygen saturation < 90%) frequently occurring during one-lung ventilation, or excessive fluid administration (≥ 30 ml/kg).

Table 2 shows intra- and postoperative outcomes of matched cohorts in both pyridostigmine and sugammadex groups. Median hospital LOS was significantly shorter in the sugammadex group than in the pyridostigmine group (10.0 days (IQR 8.0–15.0 days) vs. 12.0 days (IQR 9.5–16.5 days) (*p* = 0.005). The incidence of atelectasis (18.1 vs. 29.9%, *p* = 0.040) was lower and Epidural PCA (70.9 vs. 86.6%, *p* = 0.004) was less used in the sugammadex group than in the pyridostigmine group. However, no differences were found regarding other postoperative complications reviewed between the two reversal agents.

**Table 2:** Intra– and postoperative outcomes of matched cohort

Variable	Pyridostigmine (*n* = 127)	Sugammadex (*n* = 127)	*p* value
Surgery time (min)	210.0 (180.0–255.0)	210.0 (180.0–252.0)	0.067
PCA			0.004
Epidural	110 (86.6)	90 (70.9)	
Intravenous	17 (13.4)	37 (29.1)	
LOS (days)	12.0 (9.5–16.5)	10.0 (8.0–15.0)	0.005
PACU stay (min)	65.0 (60.0–80.0)	60.0 (55.0–75.0)	0.290
Events in PACU			
Dyspnea	4 (3.1)	3 (2.4)	1.000
HDI	7 (5.5)	4 (3.1)	0.538
Events in Ward			
Pyrexia	38 (29.9)	28 (22.0)	0.198
Dyspnea	17 (13.4)	10 (7.9)	0.222
Air leak > 5 days	27 (21.3)	26 (20.5)	1.000
Atelectasis	38 (29.9)	23 (18.1)	0.040
Pneumonia	9 (7.1)	6 (4.7)	0.594
MV	3 (2.4)	2 (1.6)	1.000
HDI	7 (5.5)	1 (0.8)	0.072
ICU	5 (3.9)	2 (1.6)	0.443

Data are presented as n (%), median (interquartile range)

*PCA* Patient controlled analgesia, *LOS* Length of stay, *PACU* Post anesthesia care unit, *HDI* Hemodynamic instability, *MV* Mechanical ventilator use, *ICU* Intensive care unit admission

Intra- and postoperative outcomes of matched cohorts in patients with and without prolonged LOS are shown in Table 3. Median LOS was 1 day shorter in the sugammadex group than in the pyridostigmine group (9.0 days [IQR 7.0–10.0 days] vs. 10.0 days [IQR 9.0–12.0 days], *p* = 0.002) in patients without prolonged LOS, whereas it did not differ between the two groups in patients with prolonged LOS (19.0 days [IQR 16.0–23.0 days] vs. 19.0 days [IQR 16.0–26.0 days], *p* = 0.537). And, PACU stay time was significantly shorter when reversed with sugammadex than with pyridostigmine (60.0 vs. 70.0 min, *p* = 0.007) in patients with prolonged LOS. Among postoperative complications, the incidence of atelectasis was significantly lower in the sugammadex group than in the pyridostigmine group (11.8 vs. 25.3%, *p* = 0.034) in patients without prolonged LOS, but did not differ between the groups (35.3 vs. 38.6%, *p* = 0.947) in patients with a prolonged LOS.

**Table 3:** Intra– and postoperative outcomes of matched cohort with and without a prolonged length of stay

	LOS ≤ 14 Days			LOS > 14 Days		
	Pyridostigmine (*n* = 83)	Sugammadex (*n* = 93)	*p* value	Pyridostigmine (*n* = 44)	Sugammadex (*n* = 34)	*p* value
Surgery time (min)	210.0 (180.0–240.0)	205.0 (175.0–240.0)	0.230	240.0 (210.0–270.0)	235.0 (185.0–255.0)	0.457
PCA						
Epidural	72 (86.7)	62 (66.7)	0.003	38 (86.4)	28 (82.4)	0.865
Intravenous	11 (13.3)	31 (33.3)		6 (13.6)	6 (17.6)	
LOS (days)	10.0 (9.0–12.0)	9.0 (7.0–10.0)	0.002	19.0 (16.0–26.0)	19.0 (16.0–23.0)	0.537
PACU stay (min)	60.0 (55.0–75.0)	60.0 (55.0–75.0)	0.611	70.0 (60.0–90.0)	60.0 (55.0–75.0)	0.007
Events in PACU						
Dyspnea	1 (1.2)	2 (2.2)	1.000	3 (6.8)	1 (2.9)	0.801
HDI	4 (4.8)	3 (3.2)	0.878	3 (6.8)	1 (2.9)	0.801
Events in ward						
Pyrexia	21 (25.3)	16 (17.2)	0.258	17 (38.6)	12 (35.3)	0.947
Dyspnea	5 (6.0)	4 (4.3)	0.861	12 (27.3)	6 (17.6)	0.466
Air leak > 5 days	3 (3.6)	6 (6.5)	0.610	24 (54.5)	19 (55.9)	1.000
Atelectasis	21 (25.3)	11 (11.8)	0.034	17 (38.6)	12 (35.3)	0.947
Pneumonia	0 (0.0)	1 (1.1)	1.000	9 (20.5)	5 (14.7)	0.720
MV	1 (1.2)	0 (0.0)	0.954	2 (4.5)	2 (5.9)	1.000
HDI	3 (3.6)	0 (0.0)	0.206	4 (9.1)	1 (2.9)	0.526
ICU	1 (1.2)	0 (0.0)	0.954	4 (9.1)	2 (5.9)	0.921

Data are presented as n (%) or median (interquartile range)

*PCA* Patient controlled analgesia, *LOS* Length of stay, *PACU* Post anesthesia care unit, *MV* Mechanical ventilator use, *HDI* Hemodynamic instability, *ICU* Intensive care unit admission

**Fig. 2:**
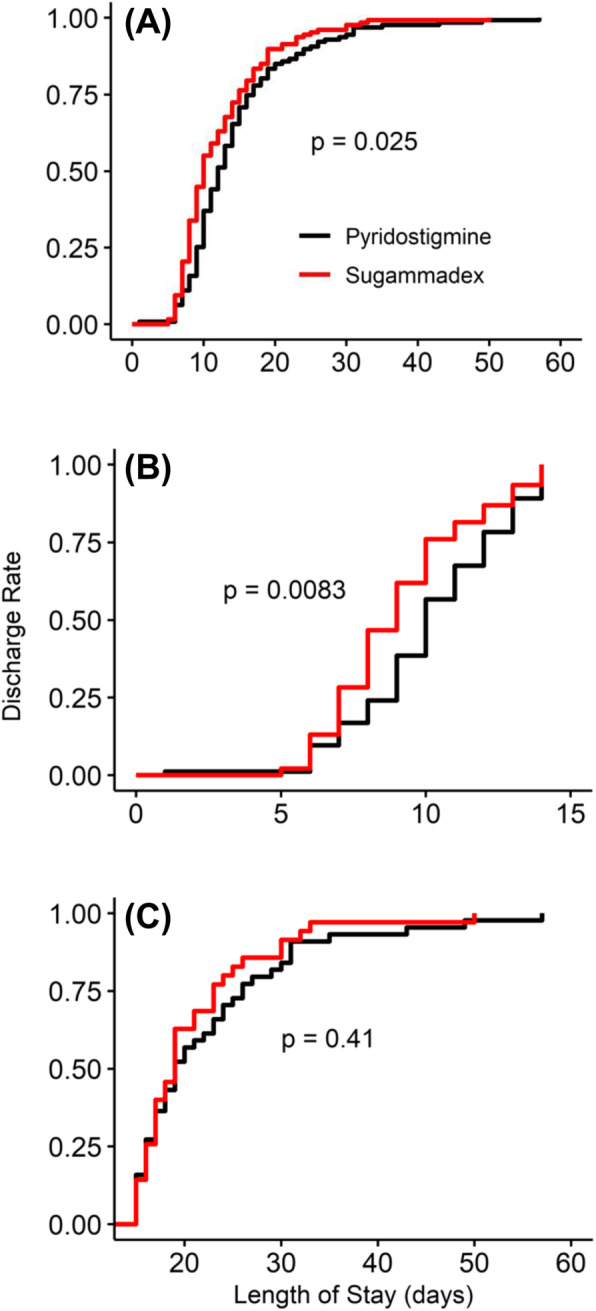
Kaplan–Meier curve for discharge in all cohorts (**a**) and in patients without (**b**) and with (**c**) prolonged hospital stay

LOS was also analyzed based on the discharge rate to determine the proportion of discharged patients on each postoperative day during hospitalization (Fig. 2). Discharge rate was significantly higher in the sugammadex group throughout the admission period (*p* = 0.025, Fig. 2a). Sugammadex also facilitated patient discharge compared to pyridostigmine in patients without prolonged LOS (*p* = 0.0083, Fig. 2b), but not in patients with prolonged LOS (*p* = 0.41, Fig. 2c).

All variables that were significant in univariate analyses were included in multivariate analyses to identify covariates associated with LOS in the propensity matched total study cohort (Table 4). In multivariate analyses, demographic predictors included four variables (age ≥ 65 years, male sex, DM, COPD). COPD and male sex showed higher risk for prolongation of LOS. Age ≥ 65 years was associated with a 9% increase in LOS. By contrast, DM reduced the LOS by 18%. The only intraoperative predictor was sugammadex, which reduced LOS by 12%. Among the postoperative factors significant in univariate analyses, dyspnea, atelectasis, pneumonia, and air leak > 5 days remained significant for increasing the risk for prolonged LOS. Pneumonia and air leak > 5 days showed a higher risk for extended LOS. Dyspnea and atelectasis were also associated with increased hospitalization.

**Table 4:** Predictors of length of stay after lobectomy for lung cancer in both sugammadex and pyridostigmine cohorts. Variables were selected from multivariable poisson regression model using forward selection and backward elimination based on the Akaike information criterion

	Exp Coef (95% CI)	***p*** value
Demographic predictors		
Age ≥ 65 yr	1.09 (1.10–1.18)	0.021
Male sex	1.27 (1.16–1.38)	< 0.001
Diabetes mellitus	0.82 (0.75–0.90)	< 0.001
COPD	1.33 (1.23–1.43)	< 0.001
Intra- or postoperative predictors		
Sugammadex (vs. pyridostigmine)	0.88 (0.82–0.95)	< 0.001
Dyspnea	1.39 (1.27–1.55)	< 0.001
Atelectasis	1.21 (1.12–1.30)	< 0.001
Pneumonia	1.77 (1.58–1.99)	< 0.001
Air leak > 5 days	1.40 (1.33–1.46)	< 0.001

*Exp Coef* Exponential coefficient, *CI* Confidence interval, *COPD* Chronic obstructive pulmonary disease

**Fig. 3:**
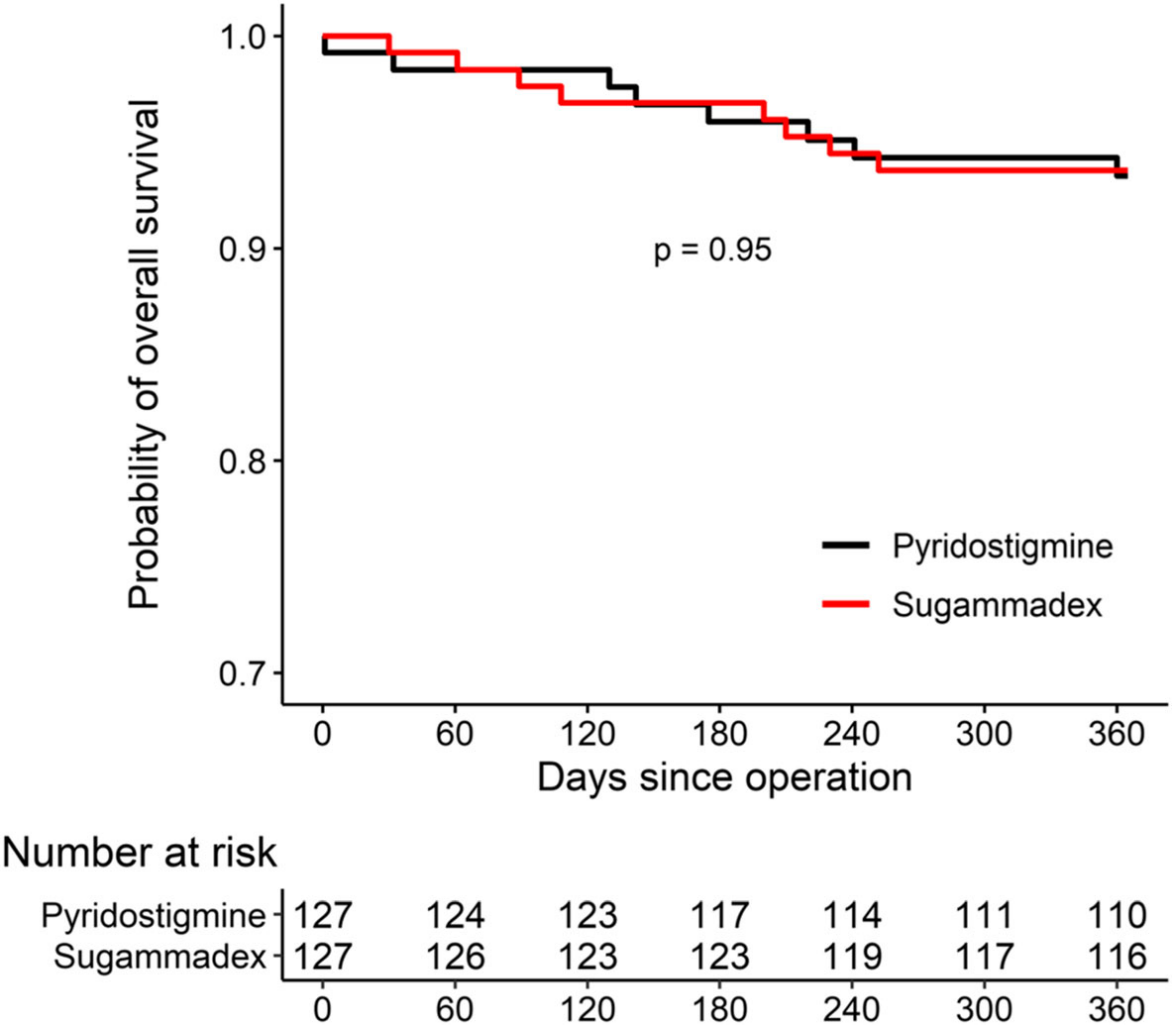
Kaplan–Meier survival estimates of death from any cause

The Kaplan-Meier curves of overall survival in the propensity-matched cohort are illustrated in Fig. 3. The estimated 1-year survival rates were 93.4% (89.1 to 97.9%) in the pyridostigmine group and 93.7% (89.6 to 98.0%) in the sugammadex group. There were no significant differences between the two groups in overall survival with an unadjusted hazard ratio for death at 1 year of 0.967; 95% CI, 0.0.363 to 2.577 (*p* = 0.947).

## Discussion

In this retrospective study, we observed a median 2.0 day decrease in time from surgery to discharge, a significantly lower postoperative complication rate (e.g., atelectasis), and a similar mortality over 1 year after the surgery when reversed with sugammadex compared to pyridostigmine. Moreover, sugammadex was the only intraoperative predictor associated with reduced LOS.

Hospital LOS was decreased, as was the incidence of adverse postoperative outcomes (e.g., atelectasis) in patients reversed with sugammadex for NMB, in line with previous studies that reported that the use of sugammadex is associated with 20% shorter LOS with reduced postoperative adverse outcomes after major abdominal surgery [12] and is associated with a 0.6 day shorter hospital LOS and a lower postoperative complication rate after laparoscopic gastric cancer surgery [18]. Residual NMB occurs in approximately 20–60% of surgical patients [23] and is associated with an increased incidence of PPCs (e.g., hypoxemia and atelectasis) [8]. On the other hand, sugammadex has been shown to reduce the incidence of residual NMB upon arrival in the PACU compared to other classic NMB reversal agents [24]. Therefore, sugammadex may decrease hospital LOS through its improved muscle relaxant reversal, leading to a reduction in PPCs and early patient discharge.

By contrast, Ledowski et al. [17] observed that overall hospital LOS after surgery did not differ between patients treated with sugammadex or acetylcholinesterase inhibitors; the cause of this discrepancy remains unclear. It has been shown that postoperative residual NMB and associated adverse PPCs are more common in elderly patients than in younger patients [25]. Moreover, thoracic and abdominal procedures that reduce lung volume are associated with increased risk of developing atelectasis and postoperative complications [26]. Ledowski et al. [17] studied relatively young patients (mean age ~ 50 years) who underwent surgical procedures including orthopedic, plastic, general, and others, whereas we studied elderly patients (mean age ~ 66 years) who underwent open lung surgery. Thus, different ages of surgical populations and type of surgery may be responsible for differences between the studies. Indeed, Ledowski et al. [17] demonstrated that NMB reversal with sugammadex significantly improves postoperative pulmonary outcomes compared to neostigmine, particularly in elderly patients.

It has previously been reported that poorly controlled acute postoperative pain is a risk factor associated with respiratory complications [27], and that postoperative pain may lead to the development of atelectasis because it can interfere with the normal activity of respiratory muscles and forced respiratory effort [28]. On the other hand, other studies have shown that epidural analgesia provides better postoperative pain control than systemic opioid administration in abdominal or open thoracotomy surgery [29, 30]. In the present study, epidural patient-controlled analgesia was used less commonly in patients reversed with sugammadex than those reversed with pyridostigmine. The postoperative pain is expected to be more severe in the sugammadex group, but the hospital LOS was short and PPCs occurred less. These findings are in accordance with those of a recent study that showed that patients reversed with sugammadex had fewer postoperative complications and a shorter LOS despite more severe postoperative pain compared to those with neostigmine in patients who had undergone laparoscopic gastric cancer surgery [18].

Several studies have reviewed patients undergoing pulmonary resection for lung cancer and identified risk factors for prolonged hospital LOS [1, 20, 28, 31–34]. Some risk factors identified previously include older age [20, 32, 33], male sex [20], ASA physical status score [20, 32], insulin-dependent diabetes [20], renal dysfunction [20], percentage predicted FEV1 [20, 33], surgeon [33], smoking [20], COPD [32], and postoperative complications (e.g., pneumonia [32, 33], unplanned reintubation [32, 34], or prolonged ventilation [32, 34]). In this study, we confirmed the important risk factors for morbidity and LOS after lung resection (Table 4). In fact, the use of sugammadex is becoming increasingly common for NMB reversal, particularly in the elderly, with the advantage that it can reverse profound NMB, although reversal agent options are currently limited by price.

Overall survival 1 year after surgery did not differ between the two reversal agents (Fig. 3). Death after lung cancer surgery may be attributable to surgery-related major complications and to cancer progression. Although sugammadex decreases the incidence of PPCs and shortened hospital LOS in the present study, this agent is unlikely to significantly reduce surgery-related major complications. In addition, we found that once a patient reached a medically stable state and was discharged, the mortality after 1 year was not different across the type of reversal agent, suggesting that an advantage for sugammadex does not extend to the long term.

This study had several limitations. First, this is a small number, single center, retrospective study that is not free from bias in selection and treatment. Not all covariates were controlled, although the demographics and clinical characteristics were balanced by propensity score matching. Second, some fundamental intra- or postoperative covariates associated with respiratory complications were not collected. The degree of pain after surgery is considered an important factor associated with respiratory complications [27]. The severity of pain and opioid consumption were not assessed in this study. In addition, the total dose of neuromuscular blocking agent administered and depth of NMB at the time of reversal were not included in our analyses. Use of single or repeated doses [35] and depth of NMB at the time of reversal [36] are important factors that affect recovery after NMB. Third, diagnosis of atelectasis was entirely dependent on plain chest radiographs, although only relatively obvious cases of atelectasis seen on plain radiography were included. However, this technique for the diagnosis of postoperative lung collapse is less sensitive than computed tomography, which was not routinely performed after open lobectomy in our hospital. Finally, the extent of surgery are strongly related to patient outcomes. However, the cohorts analyzed were a highly selective group that underwent open lobectomy for lung cancer.

## Conclusion

Compared with pyridostigmine, NMB reversal with sugammadex after open lung lobectomy for lung cancer was associated with a shorter hospital stay and a lower PPC, but with a similar mortality after 1 year. In particular, the effects of sugammadex on LOS was obvious in patients without prolonged LOS. Our data suggest that sugammadex is a preferable agent for NMB reversal than cholinesterase inhibitors in this patient population. However, further prospective, randomized, controlled, and sufficiently powered studies on larger patient populations are required.

## Data Availability

Not applicable.

## Abbreviations

PPC: Postoperative pulmonary complication
ICU: Intensive care unit
NMB: Neuromuscular blockade
LOS: Length of stay
ASA: American Society of Anesthesiologists
BMI: Body mass index
FEV1: Forced expiratory volume in 1 s
FVC: Forced vital capacity
DM: Diabetes mellitus
HTN: Hypertension
CKD: Chronic kidney disease
HF: Heart failure
CAD: Coronary arterial disease
COPD: Chronic obstructive pulmonary disease
PACU: Postanesthesia care unit
PCA: Patient controlled analgesia
SD: Standard deviation
IQR: Interquartile range
CI: Confidence interval
OR: Odds ratio
